# Water-Dependent Blending of Pectin Films: The Mechanics of Conjoined Biopolymers

**DOI:** 10.3390/molecules25092108

**Published:** 2020-04-30

**Authors:** Yifan Zheng, Aidan Pierce, Willi L. Wagner, Henrik V. Scheller, Debra Mohnen, Maximilian Ackermann, Steven J. Mentzer

**Affiliations:** 1 Laboratory of Adaptive and Regenerative Biology, Brigham & Women’s Hospital, Harvard Medical School, Boston, MA 02115, USA; yzheng@bwh.harvard.edu (Y.Z.); afpierce@bwh.harvard.edu (A.P.); willi.wagner@uni-heidelberg.de (W.L.W.); 2 Department of Diagnostic and Interventional Radiology, Translational Lung Research Center, University of Heidelberg, 69120 Heidelberg, Germany; 3 Joint BioEnergy Institute, Emeryville CA and the Environmental Genomics and Systems Biology Division, Lawrence Berkeley National Laboratory, Berkeley, CA 94608, USA; scheller@berkeley.edu; 4 Complex Carbohydrate Research Center and Department of Biochemistry and Molecular Biology, University of Georgia, Athens, GA 30602, USA; dmohnen@ccrc.uga.edu; 5 Institute of Functional and Clinical Anatomy, University Medical Center of the Johannes Gutenberg-University, 55131 Mainz, Germany; maximilian.ackermann@uni-mainz.de

**Keywords:** pectin, polysaccharide, homopolymer adhesion, fracture mechanics, scanning electron microscopy

## Abstract

Biodegradable pectin polymers have been recommended for a variety of biomedical applications, ranging from the delivery of oral drugs to the repair of injured visceral organs. A promising approach to regulate pectin biostability is the blending of pectin films. To investigate the development of conjoined films, we examined the physical properties of high-methoxyl pectin polymer-polymer (homopolymer) interactions at the adhesive interface. Pectin polymers were tested in glass phase (10–13% *w*/*w* water content) and gel phase (38–41% *w/w* water content). The tensile strength of polymer-polymer adhesion was measured after variable development time and compressive force. Regardless of pretest parameters, the adhesive strength of two glass phase films was negligible. In contrast, adhesion testing of two gel phase films resulted in significant tensile adhesion strength (*p* < 0.01). Adhesion was also observed between glass phase and gel phase films—likely reflecting the diffusion of water from the gel phase to the glass phase films. In studies of the interaction between two gel phase films, the polymer-polymer adhesive strength increased linearly with increasing compressive force (range 10–80 N) (R^2^ = 0.956). In contrast, adhesive strength increased logarithmically with time (range 10–10,000 s) (R^2^ = 0.913); most of the adhesive strength was observed within minutes of contact. Fracture mechanics demonstrated that the adhesion of two gel phase films resulted in a conjoined film with distinctive physical properties including increased extensibility, decreased stiffness and a 30% increase in the work of cohesion relative to native polymers (*p* < 0.01). Scanning electron microscopy of the conjoined films demonstrated cross-grain adhesion at the interface between the adhesive homopolymers. These structural and functional data suggest that blended pectin films have emergent physical properties resulting from the cross-grain intermingling of interfacial pectin chains.

## 1. Introduction

A variety of polysaccharide polymers have been recommended for biomedical applications including cellulose [1], alginate [2], chitin [3], agarose [4], and pectin [5]. Pectin is a potentially useful polysaccharide because of its chemical and functional properties. Chemically, commercial pectins contain primarily linear chains of homogalacturonan, a partially methyl esterified polymer of (14–)-α-d-galacturonic acid (GalA) [6], along with lesser amounts of rhamnogalacturonan [7]. Contributing to its complexity in Nature, regions of homogalacturonan are covalently linked to the branched pectic polymers rhamnogalacturonan I and rhamnogalacturonan II [8] and to arabinogalactan proteins [9]. Functionally, pectin demonstrates remarkable adhesivity to gut mucins, providing a useful mechanism for controlled oral drug delivery [10]. Pectin also binds the mesothelial glycocalyx of visceral organs [5] suggesting a potential role as a mesothelial sealant [11].

Pectins’ adjustable physical properties are particularly relevant to their biomedical applications. Pectin polymers can demonstrate markedly different functional and structural features associated with changes in temperature and water content [12,13,14]. Biomedical applications are necessarily limited to a narrow temperature range, nonetheless, polysaccharide polymers like pectin can demonstrate important alterations in their physical properties with small changes in water content [14]. The loss of water from a dispersed solution of pectins can lead to the change of the physical properties of the pectin from a viscous liquid to a soft and rubbery gel [15,16,17]—a change referred to as “gel transition.” The ongoing loss of water from the pectin gel leads to a second discrete step, so-called “glass transition,” associated with a change in the physical properties of the pectin from soft and rubbery to hard and brittle.

The effect of phase states on pectin adhesivity is an important consideration in clinical applications. Previous attempts to investigate pectin adhesivity have largely focused on mucoadhesion [18,19]. The pectin mucoadhesive process has been frequently described as involving at least two steps: (i) a contact or wetting phase that facilitates the intimate contact between polymers and (ii) the interpenetration or entanglement of the pectin polymer with mucin chains [20,21]. The phase states of the interacting polymers appear to have a significant impact on both steps. The water content of the pectin polymer not only facilitates the initial interaction between polymers by modifying the local surface energy [22], but also contributes to electrostatic and hydrophobic interactions as well as hydrogen bond formation in mucoadhesion [20].

Despite the clear relevance of water in pectin adhesion, the complexity of the biologic interface between pectin and biologic tissue has complicated the interpretation of polymer adhesion [23,24]. To clarify the effect of pectin phase states at the pectin biopolymer interface, we studied a simplified adhesion system; namely, pectin polymer-polymer (homopolymer) interactions. The physical properties of these interactions were analyzed for the influence of pectin phase states on homopolymer adhesion.

## 2. Results

### 2.1. Gel and Glass Phase Film Adhesion

**Figure 1:**
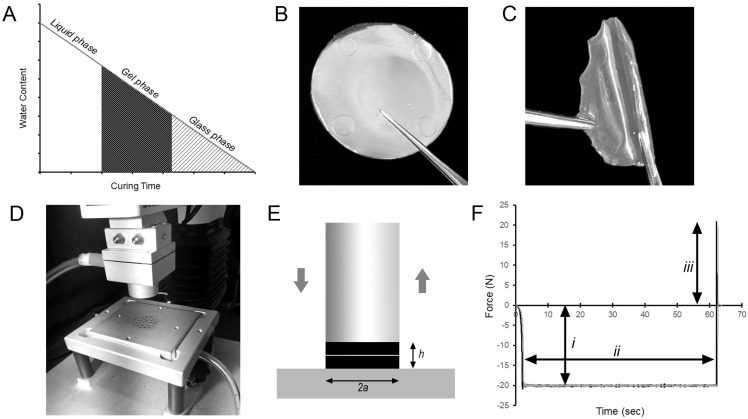
Polymer-polymer adhesion testing between glass- and gel-phase films. (**A**) Liquid pectin solutions (3% *w*/*w*) were cured in a low-humidity environment; the progressive evaporation of water created the gel phase and glass phase films used in these studies. (**B**,**C**) The films in the gel phase (38–41% *w*/*w* W_c_) were soft and flexible. The glass phase films (10–13% *w*/*w* W_c_) were hard and brittle. (**D**) Polymer-polymer adhesion experiments were performed with a custom fixture composed of a flat-ended cylindrical probe and parallel fixture surface for a preset development time. The cylindrical probe compressed the two polymers followed by the separation of the probe from the surface by an applied tensile load. (**E**) The fixture ensured that the radius of contact was defined as the radius of the probe (*a*) and the thickness of the contacted polymers was *h*. The pre- and post-experimental geometries were nearly equivalent for large values of *a*/*h* with minimal edge effects. (**F**) The data from a standard adhesion test reflected the compression force applied by the probe (*i*), the development time (*ii*) and the strength of the polymer-polymer adhesion during probe separation (*iii*).

Pectin films were cured in a controlled humidity microenvironment. In low humidity, the progressive loss of water content (W_c_) of the pectin resulted in the transition of the liquid pectin into gel phase (soft, rubbery) and subsequently glass phase (hard, brittle) films (Figure 1).

**Figure 2:**
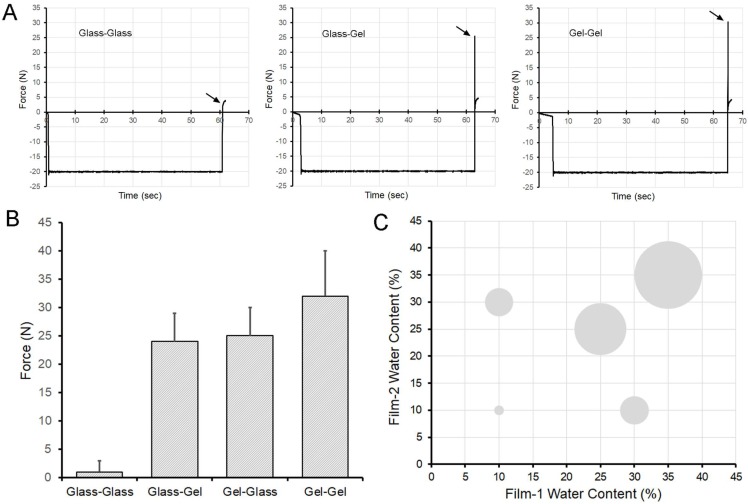
Adhesive strength of glass and gel phase films. (**A**) Glass films (10–13% *w*/*w* W_c_) demonstrated virtually no adhesion to other glass films. Gel phase films (38–41% *w*/*w* W_c_) demonstrated greater adhesion when paired with either a glass phase film or another gel phase film. Representative data is shown; arrows demonstrate peak adhesion. (**B**) The adhesion strength of glass-glass films was significantly less than gel-glass or gel-gel interactions (*p* < 0.0001) (N = 40 films; error bars ± 1 SD). Both orientations of the gel-glass and glass-gel films in the adhesion test fixture were evaluated to control for fixture or gravity influences. (**C**) The area of the bubbles reflects relative adhesion strength of the different mixtures scaled to 100.

The adhesivity of two glass phase films (glass-glass) was less than 5 N (Figure 2A). The adhesivity of one glass phase and one gel phase films (glass-gel) was 25 ± 3 N (Figure 2A). The adhesivity of two gel phase films (gel-gel) was 30 ± 3 N (Figure 2A). The adhesive strength of glass-glass films was significantly less than the adhesive strength of glass-gel or gel-gel films (*p* < 0.0001) (Figure 2B,C).

### 2.2. Polymer-Polymer Water Interdiffusion

**Figure 3:**
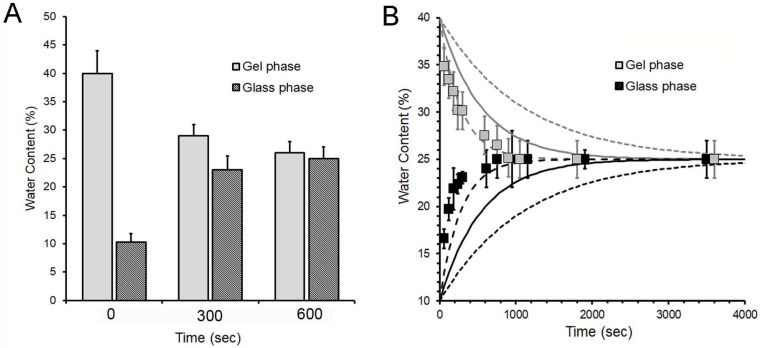
Water diffusion between pectin films. The diffusion of water was assessed between adherent gel-phase films (40% *w*/*w* W_c_) and glass-phase films (10% *w*/*w* W_c_). (**A**) Water content was assessed at time = 0, t = 5 min and t = 10 min. Each column represents the mean water content of 4 films ± 1 SD. (**B**) Water content was assessed at various intervals from t = 0 to t = 1 h. Each data point represents the mean water content of four films ± 1 SD. Since the diffusion coefficient of water through the pectin medium at 25 °C is unknown, the data is plotted relative to theoretical diffusion coefficients of 0.00092 mm^2^/s (dashed line), 0.00183 mm^2^/s (solid line) and 0.00732 mm^2^/s (dotted line) for comparison.

To assess the potential diffusion of water between a gel phase film (40 ± 3% W_c_) and a glass phase film (11 ± 2% W_c_), the films were compressed, then separated at various time intervals after initial contact. The water content was assessed by film weight. The standard glass and gel phase films reached a water equilibration within 10 min (Figure 3A). Since the diffusivity of water through the pectin medium is unknown, we plotted the empirical diffusion data against theoretical plots reflecting a range of diffusion coefficients at 25 °C. The diffusion coefficient of water through the pectin films was estimated to be 2.5- to 5-fold slower than the self-diffusion coefficient of water (Figure 3B). 

### 2.3. Compression and Development Time

**Figure 4:**
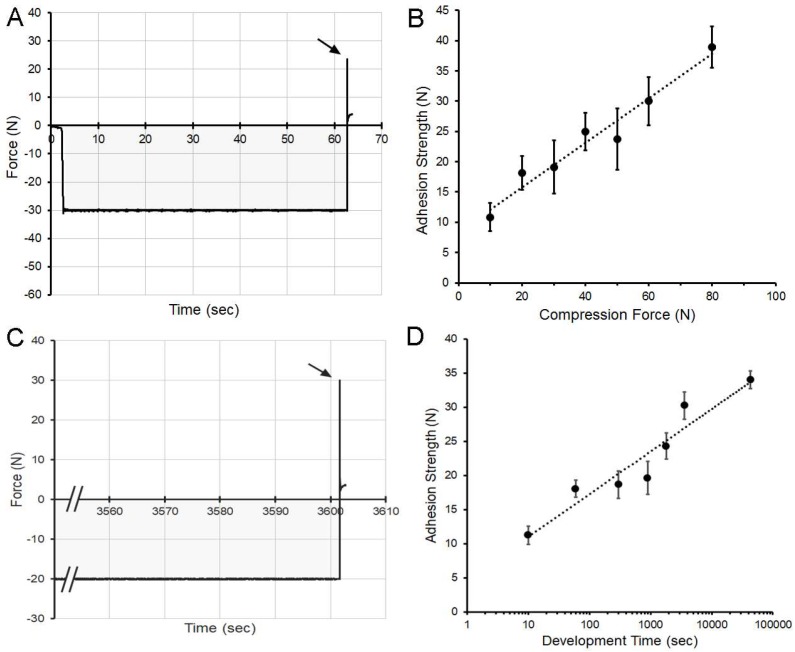
Effect of polymer-polymer compression and development time on adhesion strength. (**A**,**B**) Paired gel phase films were compressed for 60 s at compression forces ranging from 10 N to 80 N. (**A**) Representative data is shown for gel phase films compressed at 30 N. The integral of the compressive force over time (impulse) is shaded gray for presentation purposes. (**B**) The adhesive strength increased with increasing compression force. The data reflect the adhesion strength in mean N ± 1 SD (N = 70 films). A linear curve fit (R^2^ = 0.956) is shown (dotted line). (**C**,**D**) To evaluate the effect of time on polymer-polymer adhesion strength, paired gel phase polymers were compressed at 20 N for time intervals ranging from 10 s to 12 h. The films were maintained in a controlled humidity microenvironment to maintain polymer hydration. (**C**) Representative data demonstrating 20 N compression for 3600 s (1 h) followed by probe withdrawal (arrow). (**D**) Most of the adhesive strength increase was observed within 10 min of contact. The data reflect the adhesion strength in N ± 1 std. dev. (N = 45 films). A logarithmic curve fit (R^2^ = 0.913) is shown (dotted line).

The effect of polymer-polymer compression on adhesivity was assessed using an adhesion assay with two gel phase polymers. The compressive force, varied from 10 N to 80 N, was applied for 60 s (Figure 4A,B). The adhesive strength increased with increasing compression (R^2^ = 0.956) (Figure 3B). To study the influence of development time on polymer-polymer adhesion, a controlled humidity microenvironment was used to maintain polymer water content over time. Gel phase films were compressed with a force of 20 N for time periods varying from 10 s to 12 h (Figure 4C,D). The compression time and adhesive strength demonstrated a logarithmic relationship (R^2^ = 0.913) with most of the adhesive force developing within minutes of contact (Figure 4D).

### 2.4. Polymer-Polymer Pectin Interdiffusion

**Figure 5:**
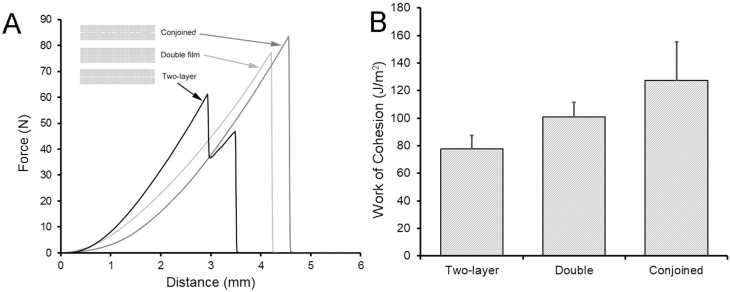
Fracture mechanics of pectin polymer-polymer films. The fracture mechanics of two adherent gel phase films (conjoined) was compared to two stacked films (two-layer) and a cured film with double pectin content (double). The films in all three conditions had virtually-identical pectin content (mean 88 ± 1 mg) and water content (mean 13 ± 0.4%) when fractured. (**A**) The pectin films were loaded (1 mm/s) with a 5-mm spherical stainless-steel probe until fracture. The peak force was recorded as burst strength. The two-layer films typically demonstrated a bimodal pattern consistent with independent fracture of the two stacked films. Burst strength, reflecting the peak force required for fracture, was not significantly different between the three films (*p* > 0.01). (**B**) The decreased stiffness and increased extensibility of the conjoined films was associated with a significantly increased work of cohesion (J/m^2^) (*p* < 0.01).

The fracture mechanics of three types of films were studied—each with comparable pectin (88 ± 1 mg) and water (13 ± 1%) content: (1) conjoined films produced by compression (10 s at 10 N) of two gel phase films followed by curing to glass phase; (2) double-thickness films produced by high-shear mixing and curing to glass phase; (3) two-layer films produced by stacking single-layer glass phase films (Figure 5). Fracture mechanics were assessed using a constant velocity uniaxial load applied normal to the plane of the polymer film until fracture. The conjoined and double-thickness films produced a single fracture peak, but the two-layer films demonstrated a bimodal fracture pattern suggesting the independent fracture of the two stacked films (Figure 5A). Notably, the burst strength of conjoined films (82 ± 14 N), double-thickness films (71 ± 12 N) and two stacked (two-layer) films (70 ± 16 N) was similar (*p* > 0.01). In contrast, the conjoined films demonstrated decreased stiffness (conjoined 19.4 ± 4.1 N/mm; double, 28.3 ± 1.6 N/mm; two-layer, 26.3 ± 0.9 N/mm) and increased extensibility (conjoined, 4.3 ± 0.5 mm; double, 3.1 ± 0.2 mm; two-layer, 3.3 ± 0.3 mm) (*p* < 0.01). Consistent with these findings, the area under the fracture curve, reflecting the work of cohesion, was 30% greater in the conjoined films than the double-thickness or two-layer films (Figure 5B) (*p* < 0.01).

### 2.5. Scanning Electron Microscopy

**Figure 6:**
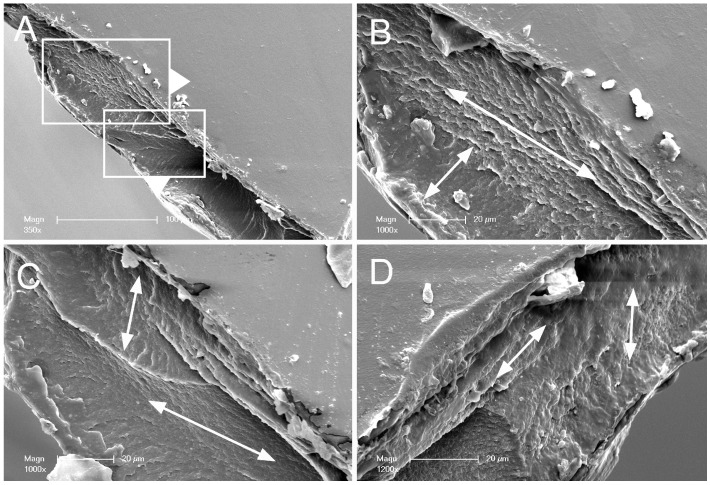
Scanning electron microscopy (SEM) of pectin polymer-polymer films. Conjoined films were produced by compressing two gel phase films at 20 N for 60 s followed by curing to the glass phase. The glass phase conjoined films were sharply divided at the midpoint and examined by SEM. (**A**) A polymer-polymer interface is at the cut surface. (**B**–**D**) High resolution images of the conjoined films. Evidence for a cross-grain effect is illustrated by the arrows.

To investigate the structural basis for these cohesive properties, the physical interface of the conjoined films was evaluated by SEM. Conjoined films were produce by compression (60 s at 20 N) followed by dehydration to the glass phase. The films were sharply bisected and examined by SEM (Figure 6). The interface between the two conjoined films suggested a gross-grain texture (Figure 6B–D).

## 3. Discussion

Biodegradable pectin polymers have been implicated in a variety of biomedical applications ranging from the delivery of oral drugs to the repair of injured visceral organs. A promising approach to regulate pectin biostability is the blending of pectin films. To investigate the development of conjoined films, we examined the physical properties of high-methoxyl pectin polymer-polymer (homopolymer) interactions at the adhesive interface. We have made several empirical observations. (1) Water content greater than 10–13% (*w*/*w*) was required for pectin polymer-polymer adhesion. (2) Adhesion occurred rapidly (minutes) above a minimum compression threshold. (3) Polymer-polymer adhesion produced a conjoined film with distinctive cohesion properties. (4) SEM demonstrated evidence of superficial pectin bridging and cross-grain adhesion of the conjoined polymers. We conclude that pectin polymer-polymer adhesion involves a process of superficial intermingling of pectin chains that is facilitated by intimate contact and water diffusion.

Our experimental conditions were designed to simulate the adhesive interactions encountered in biomedical applications; namely, the polymer interactions were studied in air and with a water content restricted to glass and gel phase polymers. We recognize that there are intriguing conceptual issues that remain undefined. First, the water content at the polymer interface may differ from the water content of the bulk film. Whereas this difference may influence theoretical issues of surface contact, polymer deformation and chain mobility, the reproducibility of our adhesion studies suggests that these issues are of limited practical significance. Second, pectin is a negatively charged polyelectrolyte potentially influenced by salt concentration. We restricted our study to water and a meticulously maintained humidified microenvironment. Although the influence of ions on pectin adhesion is relevant and should be considered in future studies, our recent studies indicate that phase state is the dominant predictor of both physical properties [25] and polymer-polymer adhesion.

The distinctive feature of the initial contact or wetting phase of polymer-polymer interaction is the rapidity of both adhesion and deadhesion. Adhesion occurred within seconds of contact; similarly, deadhesion occurred suddenly—producing the near-vertical deadhesion curve observed in the tensile strength assay. This water-dependent adhesion likely involves hydrogen bonding, the hydrophobic interaction between methyl groups and electrostatic forces between polymer chains. Although our SEM studies demonstrated evidence of pectin chains traversing the interface between conjoined films, we interpret these images as reflecting the superficial intermingling, rather than the substantive interpenetration, of pectin chains. We anticipate that more substantial chain interpenetration would produce effacement of the polymer interface as well as a more protracted debonding curve.

The adhesion between two gel-phase pectin polymers not only occurred rapidly, but the strength of adhesion increased with increasing compression. The progressive development of adhesive strength over time suggests the consolidation of the physico-chemical interactions involved in water-dependent adhesion. Similarly, the compression forces may have contributed to inter-polymer consolidation by reducing the distance between interacting polymers.

An unexpected finding was the cohesive strength of the conjoined films. Two compressed gel-phase (conjoined) films cured to glass phase had distinctive fracture mechanics; that is, the conjoined films had greater cohesive strength than comparable double thickness or stacked films. Although the mechanism is unclear, we speculate that this fracture resistance or toughness is a cross-grain effect [26,27]. In this interpretation, the mechanical stress produced a crack that spanned one layer, but the propagation of the crack was constrained by the conjoined layer. The bond between the conjoined films was crucial as two-layers of stacked films demonstrated a different (bimodal) fracture pattern and significantly lower work of cohesion. Similarly, a double thickness film, likely demonstrating uniform polymer orientation in the glass phase [14], also demonstrated lower work of cohesion. These results suggest that a random orientation of conjoined films—bonded at the polymer-polymer interface—produced this cross-grain effect and the emergent physical properties of the blended films.

## 4. Materials and Methods

### 4.1. Pectin

The citrus pectins were obtained from a commercial source (Cargill, Minneapolis, MN, USA) as previously described [14]. Briefly, the proportion of galacturonic acid residues in the methyl ester form determined the degree of methoxylation. High-methoxyl pectins (HMP) were defined as those pectin polymers with a greater than 50% degree of methoxylation (Mean = 66 ± 9%). The mean molecular weight was 265 kD, intrinsic viscosity was 621 mL/g, and the polydispersity index 1.84. The pectin powder was stored in low humidity at 25 °C.

### 4.2. Pectin Dissolution in Water

The pectin powder was dissolved at 25 °C by a gradual increase in added water to avoid undissolved powder as previously described [28]. Swelling and softening of the particles was followed by fluidization; complete dissolution of the pectin was achieved by a high-shear 10,000 rpm rotor-stator mixer (L5M-A, Silverson, East Longmeadow, MA, USA). The dissolved pectin (3% *w*/*w*) was poured into standard molds for further studies. The thickness of the films (h) varied with water content and amount of pectin: 40 ± 0.4 um thick in glass phase (12% water content) and 62 ± 0.6 um thick in gel phase (40% water content).

### 4.3. Humidification Chamber

A custom designed 5.7 L translucent polycarbonate humidification chamber was used to maintain stable humidity during prolonged testing as previously described [29]. Humidification was produced by an ultrasonic humidifier or manual aerosol device. The chamber was monitored by wireless (Bluetooth) hygrometer and thermometer sensors (SensorPush, Brooklyn, NY, USA). The data recording device was maintained within the humidification chamber throughout each experiment. The instrument was designed to be compatible with the AT-XT plus instrument (Stable Micro Systems, Godalming, Surrey, UK),

### 4.4. Adhesion Testing

Polymer-polymer adhesion experiments were performed with a force-calibrated custom fixture designed for the TA-XT plus with 50 kg load cell (Stable Micro Systems, Godalming, Surrey, UK, Figure 1). The fixture was composed of a 30 mm diameter flat-ended cylindrical probe and a flat fixture surface designed with vacuum fixation. The films were attached to polychloroprene mount using proprietary 3M adhesive (3M, St. Paul, MN, USA) or cyanoacrylate adhesive (VetBond, 3M, St. Paul, MN, USA). The cylindrical probe compressed the two polymers followed by the separation of the probe from the surface by an applied tensile load. The radius of contact was defined as the radius of the probe (*a*) and the thickness of the contacted polymers was *h*. The pre- and post-experimental geometries were nearly equivalent for large values of *a*/*h* with minimal evidence of edge effects. Probe velocity, compression force and distance were recorded at 500 pps. A minimum of N = 10 films per data point were tested.

### 4.5. Fracture Mechanics

To determine the fracture mechanics of the pectin, the biopolymers were subjected to a controlled uniaxial load normal to the plane of the polymer film as previously described [14,25]. Briefly, a 5 mm stainless steel spherical probe was mounted to a 50 kg load cell and positioned centrally over the biopolymer. The probe descended at a velocity of 1 mm/s until contact with the film. At a 0.049 N trigger force, then a probe velocity increased to 2 mm/s until fracture. The fracture force and distance were recorded at 500 pps. Burst strength was defined as the peak force required for film fracture (N). The distance the probe traveled between polymer contact (detection at 1 N) and film fracture was defined as extensibility (mm). The slope of the initial linear portion of the burst curve was defined as stiffness (N/mm).

### 4.6. Scanning Electron Microscopy

After coating with 20–25 A gold in an argon atmosphere, the pectin films were imaged using a Philips XL30 ESEM scanning electron microscope (Philips, Eindhoven, The Netherlands) at 15 keV and 21 μA. Images were obtained using a eucentric sample holder using standardized automation.

### 4.7. Statistical Analysis

The statistical analysis was based on measurements in at least three different samples. The unpaired Student’s t test for samples of unequal variances was used to calculate statistical significance. The data was expressed as mean ± one standard deviation (SD). The significance level for the sample distribution was defined as *p* < 0.01.

## Abbreviations

HMP	high-methoxyl pectin
kD	kilodaltons
LMP	low-methoxyl pectin
Pps	points per second
SEM	scanning electron microscopy
SD	standard deviation
W_c_	water content
